# Solid Organ Abscess by Melioidosis: An Emerging Infection Among Diabetics in Odisha, India

**DOI:** 10.7759/cureus.32975

**Published:** 2022-12-26

**Authors:** Pallabi Dash, Sandeep Kumar Prusty, Sidharth S Pattnaik, Niranjan Mohapatra

**Affiliations:** 1 Internal Medicine, Srirama Chandra Bhanja (SCB) Medical College and Hospital, Cuttack, IND

**Keywords:** odisha hidden hotspot, diabetes, solid organ abscess, melioidosis, burkholderia pseudomallei

## Abstract

Melioidosis is an infection in tropical regions, caused by Gram-negative bacillus *Burkholderia pseudomallei *and carries very high case fatality rates. Diabetes mellitus is a major risk factor for melioidosis, and Odisha with its current trends in urban prevalence of diabetes and a tropical coastal climate seems to be emerging as a hub of melioidosis.

We herein describe two cases of melioidosis encountered within a month. Case 1 is a 35-year-old male recently diagnosed with diabetes, presenting with fever and altered sensorium for 5 days. Case 2 is a 40-year-old female who complained of fever, cough, and generalised weakness for 2 months, and was subsequently found to be diabetic on routine investigations. Both the patients were subjected to imaging modalities which showed solid organ (liver and spleen) abscesses. On further investigations, *Burkholderia pseudomallei* was isolated from their blood cultures.

Diagnosis of such cases within a short period of time points to the fact that Odisha indeed serves as a hidden hotspot of melioidosis. The case report throws light on the importance of early diagnosis and treatment of melioidosis, which is under-reported in the state, and it also aims to impress upon physicians to have a strong clinical suspicion so as to decrease the mortality associated this neglected tropical disease.

## Introduction

The hyperglycemic environment in diabetes makes the patient liable to an array of infections, including abscesses in solid organs like the liver, kidney, and spleen. Melioidosis is one such systemic infection caused by a saprophytic Gram-negative, motile bacillus with bipolar staining called *Burkholderia pseudomallei* which is endemic in South East Asia and Australia [1]. It has been an emerging etiology of solid organ abscesses in diabetes, which might be attributed to blunted cellular responses to *Burkholderia pseudomallei *during acute infection in diabetes. Such poor immune responses are due to several reasons that include the following [2]: (1) decreased ability of macrophages to phagocytose and kill the bacteria; (2) decreased generation of CD4+ regulatory T (T reg) cells induced by lipopolysaccharide; and (3) disablement of myeloid differentiation primary response protein MyD88 inflammatory signalling, mediated by Toll-like receptor.

This organism is commonly found in the rhizosphere and surface groundwater of many tropical and subtropical countries, and it can survive in extreme situations such as distilled (nutrient-free) water for up to 16 years or in desert habitats [2]. Most cases (75-85%) occur during the rainy season, with occasional seasonal outbreaks.

The bacteria can be acquired via percutaneous inoculation, aerosol inhalation, and ingestion and may subsequently cause an acute, chronic, or latent disease, although, in most immune-competent hosts, it results in subclinical disease. Clinically melioidosis could be a localised disease or could result in a disseminated disease. Disseminated disease can manifest as pneumonia, abscesses in the liver, spleen, prostate, kidney, skin, and subcutaneous tissue, fever of unknown origin, or can spread haematogenously to cause chronic suppurative infections. It may also present as suppurative parotitis, submandibular abscesses, and lymphadenitis (in children) with fever being the commonest presentation. The disease has a mortality rate reaching as high as 95% in untreated cases and a rate of 50% even after treatment with antibiotic therapy. The commonly used antibiotic regimen consists of parenteral ceftazidime as intensive therapy followed by oral trimethoprim-sulfamethoxazole as maintenance therapy [3].

India is vulnerable to melioidosis due to its favourable environment, a substantial prevalence of diabetes mellitus, and a vast rural population. Despite having such risk factors and a significant prevalence, it is under-diagnosed and under- reported. Improvement in diagnostic facilities has led to the detection of more cases of melioidosis along the states of coastal belts like Karnataka, Kerala, and Tamil Nadu, but it is not limited to that region. Studies conducted in Odisha conclude that there is a major hidden focus of melioidosis in Odisha [3-4]. Our case reports corroborate with these studies and throw light on the importance of early diagnosis and treatment of melioidosis, so as to decrease the mortality associated this neglected tropical disease.

## Case presentation

Case 1

A 35-year-old male from rural Odisha presented with complaints of fever and altered sensorium for 5 days. He was diagnosed with diabetes 20 days before presentation and was yet to start any oral hypoglycemic agents. On examination, the patient was disoriented, pale, icteric, and had tender hepatomegaly on abdominal palpation. His vitals were unstable with a fever of 101.4 ^o^F and blood pressure of 60/40 mmHg owing to septic shock. His blood glucose on day 2 of admission was 202 mg/dl. On this background, a provisional diagnosis of hepatic abscess was made and the patient was put on vasopressor support, insulin, metronidazole, and ceftriaxone.

**Figure 1:**
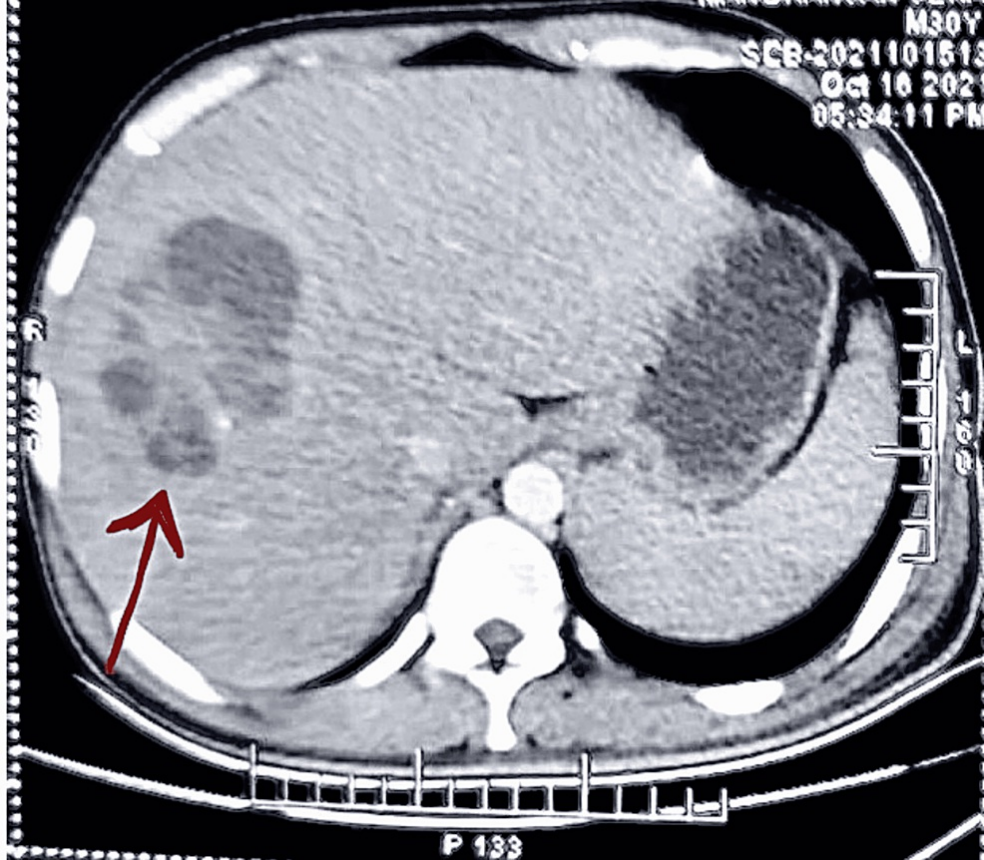
Contrast-enhanced computed tomography of the abdomen of Case 1 showing honeycomb pattern liver abscess (arrow)

On day 2, his routine investigations revealed microcytic hypochromic anemia with hemoglobin of 9.8 g/dl, a total leukocyte count of 6.662 * 10^3^/uL (neutrophil 88%, lymphocyte 10%, monocyte 1.8%, basophil 0.2%, eosinophil 0%), thrombocytopenia (total platelet count = 1.03 * 10^3^/uL), and deranged liver function tests (total bilirubin 1.7 mg/dl, direct bilirubin 1.3 mg/dL, aspartate aminotransferase (AST) 302 IU/L, alanine aminotransferase (ALT) 167 IU/L, alkaline phosphatase (ALP) 460 IU/L) but normal renal function (urea 32 mg/dL, creatinine 0.8 mg/dL). His glycated hemoglobin (HbA1c) was 7.5%, pointing towards poor blood glucose control. On day 6, an ultrasound of his abdomen suggested an enlarged liver and spleen with multiple septate cystic masses, which was further confirmed to be an abscess in contrast-enhanced computed tomography (CECT) of the abdomen on day 8, which showed honeycombing in the liver abscess (Figure 1). By day 8, the patient was afebrile but adequate glycemic control was not achieved. Moreover, there was no improvement in his liver function tests, hence the antibiotics were switched to meropenem and teicoplanin. Further, a blood culture sent on day 3 had also come negative on day 6. But due to the deteriorating condition of the patient, a subsequent blood culture was sent on day 7, which came positive for *Burkholderia pseudomallei *on Day 10, and was sensitive to ceftazidime, imipenem, and co-trimoxazole. By day 10, the sensitive antibiotics were started and the patient was shifted to the intensive care unit. But despite all standard clinical protocols, the patient succumbed 10 days after the diagnosis was made.

Case 2

A 40-year-old female from rural Odisha presented with complaints of intermittent fever associated with chills and rigor for 2 months, dry cough for 2 months and generalised weakness for 10 days. On examination, the patient was conscious with systemic examination essentially normal, but her blood pressure was too low to be recordable and a blood glucose test at admission came out to be 452 mg/dL, suggesting the provisional diagnosis as diabetes with a hyperglycemic hyperosmolar state in septic shock. The patient was accordingly put on treatment with vasopressor support, insulin, and piperacillin + tazobactam.

On day 2 of admission, her routine investigation came out as follows: a microcytic hypochromic anemia of Hb 6.6 g/dL, a total leukocyte count of 8.72 * 10^3^/microlitre (neutrophil 85%, lymphocyte 13%, monocyte 1.8%, basophil 0.2%, eosinophil 0%), thrombocytopenia (total platelet count = 41 * 10^3^/uL), and deranged liver function tests (total bilirubin 1.0 mg/dl, direct bilirubin 0.9 mg/dL, AST 715 IU/L, ALT 190 IU/L, ALP 330 IU/L) but normal renal function (urea 15 mg/dL, creatinine 0.4 mg/dL). Her glycated hemoglobin (HbA1c) was 12.4%, denoting poor control of diabetes. On the following days, the foci of the infection were searched for and subsequently, the chest X-ray showed right-sided pleural effusion. The urine routine examination as well as culture was negative for any pathogen and the blood culture was sterile by day 6. Due to our recent experience with melioidosis, this patient was started on ceftazidime from day 6 and and a CECT of abdomen and a second blood culture was sent before initiation on antibiotics. The CECT again showed honeycomb abscesses on liver as well as spleen, suggesting melioidosis, which was further corroborated by blood culture, which came positive for *Burkholderia pseudomallei* on day 10 and was sensitive to meropenem and co-trimoxazole.

After initiation of the appropriate antibiotic, the patient showed significant improvement in vitals, glycemic control as well as in liver function tests until day 22, after which her condition started deteriorating with a difficulty in maintaining adequate oxygen saturation and she went into septic shock again. She was shifted to ICU, and despite adequate critical care, she succumbed on day 25 of admission.

Table 1 gives a summary of both the cases.

**Table 1:** Summary of the cases HbA1c= Glycated hemoglobin, AST= Aspartate transaminase, ALT= Alanine transaminase, ALP= Alkaline phosphatase, INR= International Normalised Ratio, HRCT= High Resolution Computed Tomography, CECT= Contrast-Enhanced Computed Tomography

Features	Reference range of investigations	Case 1	Case 2
Age		35 years	40 years
Sex		Male	Female
Address		Rural Odisha	Rural Odisha
Occupation		Teacher	Housewife
Diabetes status		Recently diagnosed; not under treatment	Diagnosed as frank diabetes during evaluation after admission
Duration of symptoms		5 days	2 months
Presentation		Fever Altered sensorium	Fever Chills and rigor Dry cough Generalised weakness
Blood glucose as indicated by HbA1c	Normal value< 5.7%	HbA1c 7.5%, euglycemic throughout	HbA1c 12.4%; difficult to control hyperglycaemia
Hemoglobin	11.0 -17.0 gm/dL	9.8 gm/dL on day 2 which gradually decreased to 6.4 gm/dl; requiring transfusion	6.6 gm/dL on day 2 ; required transfusion
Total leukocyte count	4.0-11.0 * 10^3^/ uL	Within normal range (mean count = 6.72 * 10^3^/uL)	Within normal range (mean count = 7.82 * 10^3^/uL)
Total platelet count	150-450* 10^3^/ uL	Thrombocytopenia (mean count = 90 * 10^3^/uL) ; required transfusion	Thrombocytopenia (mean count = 90 * 10^3^/uL) ; required transfusion
Renal function tests as measured by serum creatinine	Serum creatinine = 0.6-1.1 mg/dL	Within normal range (mean creatinine value = 0.7mg/dl)	Within normal range (mean creatinine value = 0.6mg/dl)
Liver function tests		Deranged	Deranged
	Total bilirubin: 0.2-1.2 mg/dl	1.7mg/dl	1.0mg/dl
	direct bilirubin: 0.1-0.4 mg/dL	1.3 mg/dl	0.9 mg/dL
	AST: < 35 IU/L	302 IU/L	715 IU/L
	ALT: < 45 IU/L	167IU/L	190 IU/L
	ALP: < 369 IU/L	460IU/L	330 IU/L
Serum Albumin	3.5 – 5.2 gm/dL	Decreased (2.4 mg/dl)	Decreased (2.3 mg/dl)
Clotting function as measured by INR	< 1.1	Deranged (INR 2.64); required FFP transfusion	Within normal range
HIV status		Negative	Negative
Chest X-Ray		Within normal range	Right sided pleural effusion
HRCT Thorax		Not done	Right sided pleural effusion with atelactesis in lower lobe
Ultrasound of Abdomen		Liver abscess; splenomegaly	Not done
CECT of Abdomen		2 abscesses in liver with the larger one showing honeycombing; one abscess in spleen	2 abscesses in liver with the larger one showing honeycombing; multiple abscesses in spleen
Blood culture		1st culture negative; second culture positive on day 10	1st culture negative; second culture positive on day 10
Antibiotics sensitivity		Ceftazidime; Co-trimoxazole	Meropenem; Co-trimoxazole
Clinical outcome		No improvement; death on Day 22	Clinical improvement till day 22; deteriorated and died on day 25

## Discussion

The present report describes our encounter with two cases of melioidosis among adult patients from rural Odisha within a span of one month - September-October - which represents the rainy season in Odisha, thus confirming the epidemiological profile of melioidosis as described in the literature [2]. The report also shows that melioidosis can either be an acute infection presenting with sepsis due to localised abscesses (Case 1), or it can be a chronic infection presenting with symptoms lasting >2 months (Case 2). Diabetes was a risk factor found in both cases. Both of them had abscesses in the liver as well as spleen, which evoked a diagnostic dilemma with differential diagnoses like hydrated cyst, infective embolism in case of infective endocarditis, and abscess due to *Klebsiella *infection in diabetes [5], which were later ruled out with imaging and blood culture reports.

In resource-poor settings like Odisha, an ultrasound of the abdomen showing multiple solid organ abscesses in a diabetic patient serves as the initial guide to diagnose melioidosis. Further confirmation is done by CECT of the abdomen showing honeycomb abscesses, which are characteristic of melioidosis [6]. Yet culture remains the mainstay of melioidosis diagnosis as bacteremia is common in cases of melioidosis.

Despite the fact that *Burkholderia pseudomallei* can grow on most common laboratory media, it is frequently misdiagnosed as a contaminant or misinterpreted as other bacteria unless lab technicians are familiar with its morphology. Because *Burkholderia pseudomallei* is never found in normal human flora, its isolation from any clinical sample should be considered a melioidosis diagnosis [2]. Collection of appropriate clinical samples for culture to laboratories is a vital step towards the identification of the bacteria because blood cultures are positive in approximately 50% of patients. Due to such low sensitivity of cultures (60%), repeating cultures (especially of blood, sputum, urine, and pus samples) should be considered in patients with a strong suspicion of *Burkholderia pseudomallei* infection, as it is not infrequent to find subsequent samples positive despite initial negative results [2]. This is the reason why we sent for second cultures despite the first culture being negative in both our cases. But this fact that second cultures came positive, which were collected after 7 days of stay in the hospital, raises a question of whether melioidosis can be a nosocomial infection.

The antibiotic susceptibility of *Burkholderia pseudomallei* is limited to ceftazidime (Case 1), meropenem (Case 2), imipenem, and co-trimoxazole [7]. The antibiotics therapy is divided into an initial intensive therapy, preferably with ceftazidime or meropenem, and a later eradication therapy with co-trimoxazole to prevent relapse. Imipenem and meropenem have the lowest minimum inhibitory concentrations against *Burkholderia pseudomallei* and have faster bacterial killing rates than ceftazidime in vitro. Therefore meropenem is recommended as the drug of choice for severe melioidosis with septic shock. Despite this, ceftazidime is the drug of choice for most melioidosis patients as a first-line treatment as there is no compelling evidence that meropenem is superior to ceftazidime in individuals who are not critically ill [2].

## Conclusions

The outcome of melioidosis remains poor despite years of clinical research. The mortality is 100% in our report. The environmental and demographic factors of Odisha in terms of rainfall and temperature, dramatic weather events like cyclones, the extent of paddy cultivation, and the percentage of population with diabetes can build Odisha as a melioidosis capital. Thus, there’s a dire necessity for the creation of awareness among clinicians and capacity building of existing laboratories for early diagnosis and management of this neglected tropical disease. Our reports simply serve as an usher for the same purpose.
